# Intrauterine growth retardation - small events, big consequences

**DOI:** 10.1186/1824-7288-37-41

**Published:** 2011-09-07

**Authors:** Taimur Saleem, Nida Sajjad, Sanna Fatima, Nida Habib, Syed R Ali, Maqbool Qadir

**Affiliations:** 1 Medical College, Aga Khan University, Stadium Road, Karachi 74800, Pakistan; 2 Department of Pediatrics and Child Health, Aga Khan University, Stadium Road, Karachi 74800, Pakistan

## Abstract

Intrauterine growth retardation refers to a rate of growth of a fetus that is less than normal for the growth potential of a fetus (for that particular gestational age). As one of the leading causes of perinatal mortality and morbidity, intrauterine growth retardation has immense implications for the short term and long term growth of children. It is an important public health concern in the developing countries. Health statistics encompassing parameters for maternal and child health in the Indian subcontinent have shown improvement in the past few years but they are still far from perfect. Maternal health, education and empowerment bears a strong influence on perinatal outcomes including intrauterine growth retardation and should be the primary focus of any stratagem targeted at reducing the incidence of intrauterine growth retardation. A concerted liaison of various medical and social disciplines is imperative in this regard.

## Background

**Figure 1:**
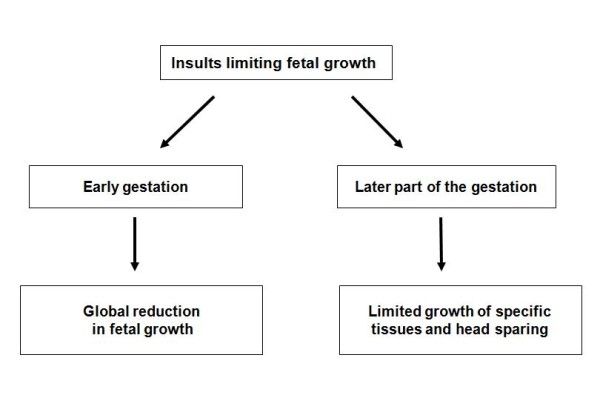
**Pattern of IUGR depends on timing of the insult during pregnancy**.

Intrauterine growth retardation (IUGR) is defined as a rate of growth of a fetus that is less than normal for the growth potential of a fetus (for that particular gestational age) [1]. It is diagnosed by two direct intrauterine growth assessments (ultra-sonographically) or when the fetal length (height) is less than two standard deviations (or third percentile) below the mean for gestational age [2]. IUGR can virtually be caused by any aberration in the normal biological processes that occur during the course of pregnancy and contribute to the growth of the fetus. It can be categorized as being either symmetric or asymmetric depending on the timing of the insult during pregnancy (Figure 1).

A fetus affected by IUGR forms a subset of cases of Small for Gestational Age (SGA) infants [3]. In SGA, the estimated weight of the fetus is below the 10th percentile for its gestational age and abdominal circumference (AC) is below the 2.5th percentile [4]. In accurately dated pregnancies, approximately 80-85% of fetuses identified as being IUGR are constitutionally small but healthy, 10-15% are 'true' IUGR cases, and the remaining 5-10% of fetuses are affected by chromosomal/structural anomalies or chronic intrauterine infections [5]. IUGR can complicate 10% to 15% of all physiologic pregnancies [6]. However, it must be remembered that the incidence of such cases varies depending on the population, geographic location being scrutinized and the standard growth curves used as reference [4].

### Sequelae of IUGR

Suboptimal fetal growth, as occurs in cases of IGUR, is an important cause of perinatal mortality and morbidity. The sequelae of IUGR include stillbirth, [3], detrimental neuro-developmental progress in childhood [3,4], higher risks of degenerative diseases in adulthood such as adult-onset diabetes, hypertension, and cardiovascular disease as well as emotional, behavioral and social problems later in life [7,8]. IUGR is also associated with significant morbidity in the form of meconium aspiration syndrome (MAS), hypoglycemia, hyaline membrane disease (HMD), early onset sepsis (EOS) and intrapartum asphyxia [4,9].

### Situation in developing countries

Prevention of low birth weight (LBW) in infants should be recognized as an important public health priority in developing countries [10]. where the condition is largely attributed to IUGR [11]. IUGR is observed in about 24% of newborns; approximately 30 million infants suffer from IUGR every year [12]. The burden of IUGR is concentrated mainly in Asia which accounts for nearly 75% of all affected infants. Africa and Latin America account for 20% and 5% cases respectively. In India, the prevalence of LBW has been reported as 26% [13]. while the proportion of IUGR has been found to be 54% [14,15]. Incidence of LBW in Pakistan has been estimated to be around 25% [12]. However, the true incidence of IUGR in Pakistan remains unknown, mainly because most of the deliveries either occur at home or the infants are not weighed after birth. In South Asia, about 74% children are not weighed at birth while in Pakistan upto 91% of the children are not be weighed at birth [16]. This may potentially underestimate the true extent and magnitude of the problem.

Observed IUGR-LBW rate in 17 datasets from developing countries compared to the incidence of IUGR-LBW estimated using the regression model shows that the incidence rate of IUGR is consistently higher than that of IUGR-LBW in all data sets by a mean difference of about 15% (95% confidence interval). The mean IUGR rate is 23.8%, ranging from 9.4% in China to 54% in India [17].

### Comparison with developed countries

A comparison of these statistics to more developed countries, such as the United States, shows that the incidence of SGA births in developed countries is about 10%. One-third of these cases represent true IUGR [18]. According to estimates, the rate of IUGR in developing countries is about 6 times higher than that in developed countries [19].

### Future directions

All the countries of South Asia are signatories to the millennium development goal (MDG) targets of reducing maternal and infant mortality by 66 - 75% by the year 2015. Paradoxically, a review of maternal and child health in South Asia has revealed that these same demographic categories of the South Asian population are neglected health-wise [20]. Given the recent progress, geopolitical inclinations and trends of investments in this area, it is not likely that these targets can be met without an intensive and holistic effort from major stakeholders including both governmental and non-governmental bodies. Experiences in the South Asian regions, such as Kerala, India and Sri Lanka, indicate that it is indeed possible to improve maternal and child health [21].

High rates of IUGR should be a cause of concern because they not only indicate an imminent risk of malnutrition and morbidity in women of childbearing age but also signal of a high risk of malnutrition, morbidity and mortality for the newborn in the developing countries [22]. A prevalence of IUGR in excess of 20% has been recommended as the cut-off point for triggering public health action. In the absence of accurate information on gestational age, a prevalence of > 15% of LBW may be used as a proxy cut-off [17]. A reduction of at least one-third in the proportion of infants with LBW is one of the seven major goals for "A World Fit for Children" programme of the United Nations [23].

Most of the data on IUGR that is available has been generated in the Western world, which may not necessarily be applicable to regions such as Pakistan and India. The genetic make-up of the South Asian population is certainly unique and has a different propensity for disease. Although the strength of the genetic basis of the risk factors for IUGR remains to be clearly elucidated, we feel that the high prevalence of the condition in the South Asian population merits well controlled, prospective studies. Concerted efforts should be made to gather indigenous data about the risk factors of IUGR that are more pertinent to our population. Evidence based recommendations deduced from such data sets are more likely to be successful and valid.

At the policy level, allocation of funding should be done for research to determine when and how the damage to the fetus can be ameliorated or prevented. Maternal risk factors such as hypertension, diabetes, tobacco and drug abuse and exposure to environmental toxins should be addressed through well planned health care interventions. Proper antenatal care should not only be made available, but easily accessible, and affordable for pregnant women in developing countries. Undoubtedly, one of the most important goals of effective antenatal care is the detection of fetuses at risk of suboptimal growth [3] at a stage where there is significant potential for remediation. It is an unfortunate instance that in the developing world, relatively few women are encountered by professional healthcare providers before they experience quickening. Therefore, a preventive management approach needs to be instituted in developing countries and this should begin with the first antenatal clinic visit. A good proportion of pregnant women attend an antenatal clinic at least once in some countries e.g., Demographic and Health Surveys show that in 30 sub-Saharan African countries, > 70% of all pregnant women attended an antenatal clinic at least once in most (22 of 30) countries [24]. At the same time, the knowledge of the health care professionals needs to be made more up-to-date to timely detect and manage IUGR and remove the risk factors for the condition from the maternal milieu.

In Pakistan and India, the problem of IUGR is compounded by poverty and insufficient per capita income. There is a lack of a clear nutritional policy along with inadequate institutional capacity and trained human resources. Large segments of the population have an inadequate dietary intake. Special attention should, therefore, be paid towards correction and prevention of maternal nutritional deficiencies. For instance, maternal dietary zinc supplements have improved fetal growth when zinc deficiency was prominent [1]. Folic acid is an important periconceptional nutrient that helps prevent neural tube defects in the fetus and megaloblastic anemia in the mother [25]. Currently it is recommended that "all women of child-bearing age take 0.4 mg of folic acid when planning a pregnancy; those women who have had a previous pregnancy affected by a neural-tube defect should take 5 mg folic acid periconceptionally, starting atleast 1 month before conception and continuing throughout the first trimester of pregnancy" [25].

Foods fortified with nutrients can be provided to pregnant women and also attempts made for a behavior change to encourage healthier eating patterns to ensure a sustained healthy pregnancy. Given the strong evidence linking higher levels of maternal education with improved child survival and nutritional status [26]. and with better nutritional status of women themselves, investment in the education of females of child bearing age is expected to have enormous dividends in health, nutrition, and development [27,28]. Empowerment of women via these channels is therefore an important component of any policy to enhance the status of women.

**Figure 2:**
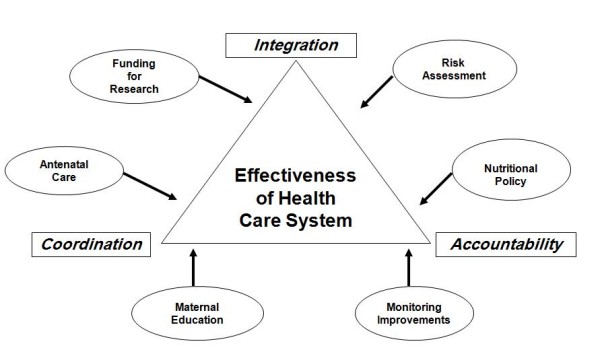
**Overview of proposed recommendations for ameliorating the incidence of IUGR**.

It should be acknowledged that the health statistics of South Asian countries have shown improvement over the past few years but they are still far from perfect. The road for the uplift of maternal and infant health remains checkered by many obstacles. Vertical programs have been run without ensuring quality of management and long term sustainability. Clarity, concision and accountability along with measurable objectives are needed for such programs. Greater integration and coordination among primary, secondary and tertiary health care facilities should also be ensured to tackle the problem (Figure 2). Undergraduate training of doctors should be revamped so as to adopt a community-based integrated approach with the inclusion of family care and counseling in the health care delivery systems. To summarize, the successful management of IUGR requires a concerted liaison of both medical and socials sectors in the developing world.

## Competing interests

The authors declare that they have no competing interests.

## Authors' contributions

TS, MQ and SRA were involved in study conception, literature search and manuscript writing. NS, NH and SF were involved in literature search and manuscript writing. All authors have read and approved the final version of the manuscript.
